# Transcriptome Profile During Rabies Virus Infection: Identification of Human CXCL16 as a Potential New Viral Target

**DOI:** 10.3389/fcimb.2021.761074

**Published:** 2021-11-05

**Authors:** Lena Feige, Inés Sáenz-de-Santa-María, Beatrice Regnault, Rachel Lavenir, Anthony Lepelletier, Ala Halacu, Randrianasolo Rajerison, Sylvie Diop, Chhor Nareth, Jean-Marc Reynes, Philippe Buchy, Hervé Bourhy, Laurent Dacheux

**Affiliations:** ^1^ Institut Pasteur, Université de Paris, Lyssavirus Epidemiology and Neuropathology Unit, National Reference Center for Rabies, WHO Collaborating Center for Reference and Research on Rabies, Department of Global Health, Paris, France; ^2^ Institut Pasteur, Université de Paris, Membrane Traffic and Pathogenesis Unit, Paris, France; ^3^ Institut Pasteur, Université de Paris, Genopole, Paris, France; ^4^ National Agency for Public Health, Chișinău, Moldova; ^5^ University Hospital Center of Befelatanana, Antananarivo, Madagascar; ^6^ Infectious Diseases Department, National and University Hospital Center of Fann-Dakar, Dakar, Senegal; ^7^ Calmette Hospital, Phnom Penh, Cambodia; ^8^ Virology Unit, Institut Pasteur de Madagascar, Tananarive, Madagascar; ^9^ Virology Unit, Institut Pasteur in Cambodia, Phnom Penh, Cambodia

**Keywords:** rabies, transcriptome, neuroinflammation, human, mouse, brain, immune response, *CXCL16*

## Abstract

Rabies virus (RABV), the causative agent for rabies disease is still presenting a major public health concern causing approximately 60,000 deaths annually. This neurotropic virus (genus *Lyssavirus*, family *Rhabdoviridae*) induces an acute and almost always fatal form of encephalomyelitis in humans. Despite the lethal consequences associated with clinical symptoms of rabies, RABV limits neuro-inflammation without causing major histopathological lesions in humans. Nevertheless, information about the mechanisms of infection and cellular response in the central nervous system (CNS) remain scarce. Here, we investigated the expression of inflammatory genes involved in immune response to RABV (dog-adapted strain Tha) in mice, the most common animal model used to study rabies. To better elucidate the pathophysiological mechanisms during natural RABV infection, we compared the inflammatory transcriptome profile observed at the late stage of infection in the mouse brain (cortex and brain stem/cerebellum) with the ortholog gene expression in *post-mortem* brain biopsies of rabid patients. Our data indicate that the inflammatory response associated with rabies is more pronounced in the murine brain compared to the human brain. In contrast to murine transcription profiles, we identified CXC motif chemokine ligand 16 (*CXCL16*) as the only significant differentially expressed gene in *post-mortem* brains of rabid patients. This result was confirmed *in vitro*, in which Tha suppressed interferon alpha (IFN-α)-induced *CXCL16* expression in human CNS cell lines but induced *CXCL16* expression in IFN-α-stimulated murine astrocytes. We hypothesize that RABV-induced modulation of the CXCL16 pathway in the brain possibly affects neurotransmission, natural killer (NK) and T cell recruitment and activation. Overall, we show species-specific differences in the inflammatory response of the brain, highlighted the importance of understanding the potential limitations of extrapolating data from animal models to humans.

## Introduction

Rabies virus (RABV) is the etiological agent of rabies, an acute and always fatal form of encephalomyelitis which can potentially affect all mammals, including humans. Even though rabies has been known and described for millennia (Baer, 1975), the physio-pathological mechanisms inducing this pathology and ultimately leading to the death of the infected individual remain unknown (Hemachudha et al., 2013). A breakthrough in eliminating rabies has been made with the introduction of rabies vaccination by Louis Pasteur in the late 1880s (Baer, 1975), opening the way to effective pre- and post-exposure prophylaxis protocols which are nowadays available (WHO, 2018; O’Brien and Nolan, 2019). However, once symptoms appear, no effective human treatment exists so far, resulting in a global burden of estimated 60,000 human deaths per year, more particularly in low-and-middle income countries in Asia and Africa (Hampson et al., 2015; Fooks et al., 2017).

Human rabies is a zoonosis, mainly transmitted after exposure to saliva of infected dogs through bites, scratches, or licking of wounds, or mucosa (Fooks et al., 2014). Upon the bite, it is assumed that RABV replicates locally in the wound, before spreading from the neuromuscular junction to motor neurons to reach the peripheral nervous system (PNS) (Murphy and Bauer, 1974; Watson et al., 1981; Davis et al., 2015). Subsequently, RABV is transported retrogradely from the PNS to the central nervous system (CNS) *via* trans-synaptic spread between connected neurons (Ceccaldi et al., 1989; Ugolini, 2011). At this stage, the infection will lead to rabies, an acute and always fatal form of encephalomyelitis with two major clinical forms: encephalitic (or furious) rabies associated with phobic (hydrophobia or aerophobia) or inspiratory spasms, autonomic stimulation signs and fluctuating consciousness, or paralytic (or dumb) rabies characterized by an ascending paralysis which can be mistaken with Guillain-Barré syndrome (Hemachudha et al., 2002; Hemachudha et al., 2013). The virus then disseminates to innervated organs or tissues as well as salivary glands through centrifugal (anterograde) propagation *via* sensory innervations (Jackson et al., 1999).

Neurotropic RABV presents a unique pathogen which is particularly well adapted to infect the CNS by preventing neuronal apoptosis and inhibiting the host immune response, two important factors that shape RABV immune evasion (Lafon, 2008; Davis et al., 2015). Although IFN-antagonistic functions of RABV genes are well studied *in vitro* and *in vivo* (Ito et al., 2016), data on RABV-mediated modulation of signalling pathways in the human CNS are still lacking. Further, increasing evidence points out major discrepancies between human and murine immune systems such as different inflammatory cell subsets, toll-like receptors (TLRs), cytokines and cytokine receptors, chemokines and chemokine receptors, as well as costimulatory signals (Mestas and Hughes, 2004). Although animal models are informative to study virus immunopathology (Louz et al., 2013), species-specific features reveal extensive differences between proportions of neuronal cell type populations, laminar distributions, gene expression, and cell morphologies between the human and murine cortex (CX) (Hodge et al., 2019) questioning if data acquired from studying neurotropic infections in murine models can be entirely extrapolated to humans.

In mice, infection with wildtype (wt) RABV isolates induces only limited inflammatory responses after intracerebral (ic) or intramuscular (im) inoculation compared to attenuated strains (Wang et al., 2005). Still, recent mRNA transcription profiling provides evidence that virulent RABV or other lyssaviruses induce an antiviral immune response in mice, through the differential modulation of gene expression of several pattern recognition receptors (*Tlr2*, *Tlr3*, *Ddx58*, *Ifih1*), genes of the complement pathway (*C1qa*, *C1qb*, *C1qc*, *C3*, *C4*), IFN regulatory genes (*Myd88*, *Irf1*, *Irf3*, *Irf7*, *Irf8*), chemokines (*Ccl5*, *Cxcl10*, *Ccl12*), and pro-inflammatory cytokines (*Il1β*, *Il6*, *Il12*) (Zhao et al., 2011; Koraka et al., 2018). RABV or related lyssaviruses upregulate genes involved in pyroptosis (Koraka et al., 2018), cell adhesion, blood vessel morphogenesis, and coagulation (Zhang et al., 2016), proteolysis, the complement cascade (Zhao et al., 2011), whereas genes involved in synaptic transmission and ion transport were significantly downregulated in the murine CNS (Zhang et al., 2016). In detail, the JAK-STAT (Zhao et al., 2011; Jamalkandi et al., 2016; Sonthonnax et al., 2019), RAS, PI3/AKT, TLR and NOTCH (Jamalkandi et al., 2016) signalling pathways were identified as the underlying differentially regulated pathways responsible for immuno-regulation, cell survival or apoptosis, and synaptic modulation. Additionally, RABV modulates the expression of long non-coding RNAs in mice which are supposedly implied in the host response against RABV infection (Zhao et al., 2018). Subtraction hybridization of CVS-N2c-infected mice revealed that about 90% of the genes were downregulated at day 6 of infection whereas only 1.4% of all genes were upregulated, suggesting that profound suppression of host gene synthesis might trigger neuronal dysfunction in mice (Prosniak et al., 2001; Fu and Jackson, 2005).

In humans, morphological findings are relatively mild (Fu and Jackson, 2005) apart from characteristic neural inclusion bodies, so-called Negri bodies (NBs) (Jogai et al., 2000; Jackson et al., 2001), suggesting that RABV preserves neuronal integrity to support its propagation (Jackson et al., 2008; Hemachudha et al., 2013). Although controversially discussed (Juntrakul et al., 2005), neuronal apoptosis seems not to play a major role in human rabies (Jackson et al., 2008; Fernandes et al., 2011) whereas inflammatory cells might undergo apoptosis (Suja et al., 2011). Yet, the role of apoptosis in natural RABV infection has not been elucidated, although virus-dependent and cell-dependent mechanisms may account for it (Fu and Jackson, 2005). It has been suggested that RABV evades the innate immune response of the human CNS (Lafon, 2011; Hemachudha et al., 2013), however, transcriptome data of human rabid patients are still lacking. Despite the expression of TLR3 in human neurons (Jackson et al., 2006), RABV implies TLR3 in the spatial arrangement of RABV-induced NBs (Ménager et al., 2009) possibly inhibiting the downstream induction of IFNs (Lafon, 2011). Further, immunostaining of histopathological sections of 25 brains from patients presenting the two main form of rabies (furious and paralytic rabies) showed no correlation between viral antigen distribution and IL-1β or TNF-α protein expression levels in microglia, macrophages or lymphocytes (Solanki et al., 2009). Taken together, little is known about the inflammatory response induced in the CNS of rabid patients.

Here, we investigated the expression of a panel of selected inflammatory genes upon infection with the virulent canine RABV strain Tha (cell culture-adapted virus) in the murine cortex (CX) and brain stem/cerebellum (BSC) *via* quantitative polymerase chain reaction (qPCR). To investigate if similar signalling pathways were differentially modulated in human rabies, we compared the transcriptome profiles of brains from infected mice to the transcriptome profile of *post-mortem* brain samples from RABV-infected patients. Whereas the mouse brain displayed a strong inflammatory response upon pathogenic RABV infection, expression of inflammatory genes was less modulated in human brain samples. In contrast to the murine model, we identified *CXCL16* as the only slightly differentially downregulated gene in *post-mortem* brain samples from rabid patients. Correspondingly, human and murine CNS cell types *in vitro* revealed the same discrepancy suggesting that virulent canine RABV targets the CXCL16/CXCR6 axis in a species-specific manner.

## Materials and Methods

### Patients and Brain Samples

Brain biopsies analysed in this study were obtained from two groups of patients (1 sample per patient). The first one originated from Cambodia and included 10 rabid patients infected with RABV canine strains circulating in this country and 4 non-rabid control patients (without apparent CNS disorders or infection) (**Supplementary Table S1**). The second group encompassed additional rabid patients from Madagascar (n=1, 1 sample), Senegal (n=1, 1 sample), Moldavia (n=1, 3 samples) infected with RABV spilling over from carnivores (mainly dog), as well as additional controls of non-rabies infected patients from Cambodia (n=1, 1 sample per patient, without apparent CNS disorder or infection) and France (n=4, 1-3 samples per patient, all presented encephalitis- or meningoencephalitis-related symptoms but without any determined aetiologies) (**Supplementary Table S2**). Biopsies originated from patients from Cambodia, Madagascar, and Senegal were part of a cohort already described in previous publications (Dacheux et al., 2008; Dacheux et al., 2016), which was approved by the national ethical committees. Brain biopsies which originated from French patients were received at the National Reference Centre for Rabies for diagnostic purpose and included in this study for secondary research use. Similarly, the Moldavian patient’s brain biopsies were received at the World Health Organization Collaboration Centre for Reference and Research on Rabies for diagnostic purpose and included in this study for secondary research use. In both cases, informed consent was obtained directly from patients or their relatives. A molecular diagnosis of rabies was performed (*ante-mortem* or *post-mortem*) for all patients included in this study by RT-PCR (**Supplementary Tables S1**, **S2**) (Dacheux et al., 2008).

### Mice and Infection Experiments

Six-week-old female BALB/c mice (Charles River Laboratories, France) were intramuscularly (im) infected with 200 μL (2 x 50 μL in each of hind leg) of a RABV virus suspension of 9.8x10^6^ focus forming units (FFU) per mL (approximately 2x10^6^ FFU per animal). The cell culture-adapted RABV field strain Tha (isolate 8743THA, EVAg collection Ref-SKU: 014V-02106) (Delmas et al., 2008) was used for infection experiments. This virus, closely related to the ones circulating in Cambodia (all of them belonging to the Southeast Asian 3 lineage within the Asian clade), was initially isolated from the brain of a Thai patient who died after being infected by a rabid dog. This virus was further adapted to cell culture on BSR cells (a BHK-21 clone) (Sato et al., 1977), and passage 21 was used for this study. Sequence comparison between the cell culture-adapted RABV strain 8743THA (GenBank No. EU293121) and the original field isolate Tha (8764THA, GenBank No. EU293111) demonstrated 98.56% identity (52 mutations) and 99.36% similarity between concatenated protein sequences of the different open reading frames. Food and water were given *at libitum* and mice were daily observed after infection. Animals were euthanized by dislocation of the cervical vertebrae at the paralysis stage (day 8 post-infection), then CX and BSC were collected after autopsy and used for RNA extraction. Non-infected six-week-old female BALB/c mice were used as negative controls.

At the time all animal experiments were performed according to the French legislation and in compliance with the European Communities Council Directives (N°86/609/CEE, 24-11-1986). The animal experimentation was approved (agreement A75-15-25, 30-07-2002) before experiments were initiated.

### Cells and Virus Infection

The human neuroblastoma cell line SK-N-SH (ATCC HTB-11), human astrocytic cell line SVGp12 (ATCC CRL8621), human microglial cell line HMC3 (ATCC CRL-3304) and murine neuroblastoma cell line Neuro-2a (N2A, ATCC CCL-131) were cultured in Dulbecco’s Modified Eagle Medium (DMEM) (10566016, Thermo Scientific) supplemented with 10% heat-inactivated foetal bovine serum (S182H-500, Eurobio) at 37°C and 5% CO_2_.

Murine cortical astrocytes (mAstrocytes) prepared from C57BL/6 wild type mice (in-house colony, Institut Pasteur, Paris, France) were used at P0 to P3 (1-3 days after birth), as described previously (Schildge et al., 2013). Briefly, after decapitating the pups, the meninges were stripped off the brain, and cortices were pooled prior to homogenization. Dissociated cortical cells (neocortex) were grown in 75 cm^2^ flasks coated with poly-D-lysine (A3890401, Thermo Scientific, working concentration 5 μg/mL) in complete astrocyte medium composed of DMEM supplemented with 10% heat-inactivated foetal bovine serum (S182H-500, Eurobio) and penicillin/streptomycin (15140148, Thermo Scientific). Cultures were kept at 37°C and 5% CO_2_ until they reached confluency (within 7–10 days of culture). Culture medium was refreshed every three days. To reduce the proliferation of other glial cells, 10 μg/mL 5-fluoro-2′-deoxyuridine (F0503, Sigma-Aldrich) and 10 μg/mL uridine (U3003, Sigma-Aldrich) were added to the culture for one week. Experiments were performed on cells plated at a density of 2.5x10^4^ cells/cm^2^ on 12-well-plates previously coated with poly-D-lysine. mAstrocytes were used until passage 5 for experiments, as late passages induce astrocyte phenotype changes (Bang et al., 2021). All animal experiments were performed according to the French legislation and in compliance with the European Communities Council Directives (2010/63/UE, French Law 2013–118, February 6, 2013) and according to the regulations of Institut Pasteur Animal Care Committees. The Animal Experimentation Ethics Committee (CETEA no. 089) of the Institut Pasteur approved this study (25-04-2018) before experiments were initiated.

Cells were infected with the Tha virus at a multiplicity of infection (MOI) of 5. After 24 hours, cells were stimulated using 2500 U/mL of universal IFN-α active on human and murine cells (I4401, Sigma-Aldrich). Cells were lysed 48 hours post-infection by using the RNeasy Mini Kit (74104, Qiagen).

### RNA Preparation and cDNA Synthesis

Human brain biopsies (size approximatively between 0.25 and 1 cm^3^) were washed at least two times in sterile PBS (10010023, Thermo Scientific), then total RNA was extracted using Tri-Reagent (TR118, Molecular Research Center) according to manufacturer’s recommendations. RNA was purified using the RNeasy Micro Kit (74004, Qiagen) with a DNA depletion step using DNase I together with the RNase-Free DNase set (79254, Qiagen), and following the recommendations of the manufacturer. The concentration of RNA was measured by photometric quantification using Nanodrop (Version 2000/2000c, Thermo Scientific) and the RNA integrity was evaluated using 2100 Bioanalyzer system (Agilent) which provides an RNA integrity number (RIN). To have sufficient RNA concentrations for the transcriptomic analysis of samples listed in **Supplementary Tables S1** and **S2**, an *in vitro* amplification step of RNA was performed using the GeneChip Two-Cycle cDNA Synthesis Kit (Agilent) in combination with the MEGAscript T7 Kit (Ambion), both according to the manufacturer’s recommendations, and starting from 10 ng or 100 ng of purified RNA. For additional human samples (**Supplementary Table S2**) and for mouse samples, RNA was isolated from tissues using Tri-Reagent as previously described. Quality and quantity of total extracted RNA were controlled by photometric quantification using Nanodrop, and reverse transcription was then performed using SuperScript II Reverse Transcriptase (18064022, Invitrogen) according to the manufacturer’s instructions. A quantity of 100 ng of purified RNA was converted into cDNA for all human samples, except for samples from patients IPC23 and IPC24 for whom a quantity of 10 ng was used. A total of 1 μg of purified RNA from mouse samples was used for reverse transcription. For qPCR experiments, cDNA was diluted 1:10 – 1:100 depending on the initial RNA concentration that was used for reverse transcription, and on the preparation method used.

### Quantitative PCR

A list of 33 inflammatory genes was selected, based on a preliminary transcriptome analysis conducted on a limited panel of human (n=4, 2 positive and 2 negative) and mouse samples (3 positive and 3 negative) using the Affymetrix microarrays GeneChip^®^ Human Genome U133 Plus 2.0 and GeneChip^®^Mouse Genome 430A 2.0, respectively (data not shown). Quantitative PCR based on the detection of the SYBR Green dye was performed using 5 μl of the synthesized and diluted cDNA in presence of 10 μl QuantiTect SYBR Green (204143, Qiagen) and 1 mM specific primers (in a final volume of 20 μl, 33 targeted genes), or in the presence of 3.1 μl of Power SYBR Green PCR Master Mix (Applied Biosystems, 4367659) and 0.9 mM specific primers (in a final volume of 25 μl) (**Supplementary Table S3**). Oligonucleotides were used for PCR at a concentration of 10 pmol/μl. All the samples were measured in triplicates. Gene expression levels were normalized to the endogenous expression of the geomean of two housekeeping genes, 18S ribosomal RNA (*18S*) and glyceraldehyde-3-phosphate dehydrogenase (*GAPDH*) for human samples and actin beta (*Actb*) and *Gapdh* for murine samples (**Supplementary Figure S1**). The ΔCT was calculated by subtracting the geomean of the above-mentioned housekeeping genes from the CT values of immunity genes (Vandesompele et al., 2002). Variations in gene expression were calculated as the n-fold change in expression in the tissues from the infected samples compared with the non-infected samples using the 2^-ΔΔCt^ method (Pfaffl, 2001). The ΔΔCT was calculated by normalizing the ΔCT values to the corresponding non-infected samples. The two best suitable housekeeping genes were selected based on the housekeeping genes showing the least variation between samples (**Supplementary Figure S1**). Conditions used for qPCR were the following: initial denaturation step (1x repetition, 10 min, 95°C), amplification step (40x repetitions, 15 sec 95°C, 1 min 60°C) and melting curve determination step (1x repetition, 15 sec, 95°C, 1 min 60°C, 15 sec 95°C, 15 sec 60°C).

### Immunofluorescence

Murine astrocytes were isolated as described before (Schildge et al., 2013). A total of 1x10^4^ cells/well was seeded into 96-well-plates (655086, Greiner Bio) previously coated with 5 μg/mL Poly-D-Lysine. After infection, cells were fixed using 4% paraformaldehyde fixative solution (J61984, Alfa Aesar) for 15 minutes at room temperature. Cells were washed once with PBS and permeabilized using 0.5% (v/v) Triton-X (648463, Millipore) for 10 minutes. After permeabilization, cells were stained overnight with GFAP (AB5804, Millipore) at 4°C. Cells were washed with PBS and incubated with Rabies DFA agent (5100, Chemicon) and the secondary antibody AlexaFluor 546 (A11035, Thermo Scientific) for 1 hour at 4°C. Cells were stained for 5 minutes with Hoechst (H1399, Thermo Scientific) and imaged using the Opera Phenix™ High Content Screening System (Perkin Elmer).

### Statistical Analysis

Percentages and means ± SD (standard deviation) were calculated with GraphPad Prism 9 and R (version 3.4.4). Statistical deviation and statistical significance were calculated when at least three independent experiments were conducted. Figures were generated by R (version 3.4.4), Prism 9, and Adobe Illustrator CC 2019.

## Results

### RABV Strain Tha Targets IFN-Signalling Pathways in the Murine CX and BSC

**Figure 1:**
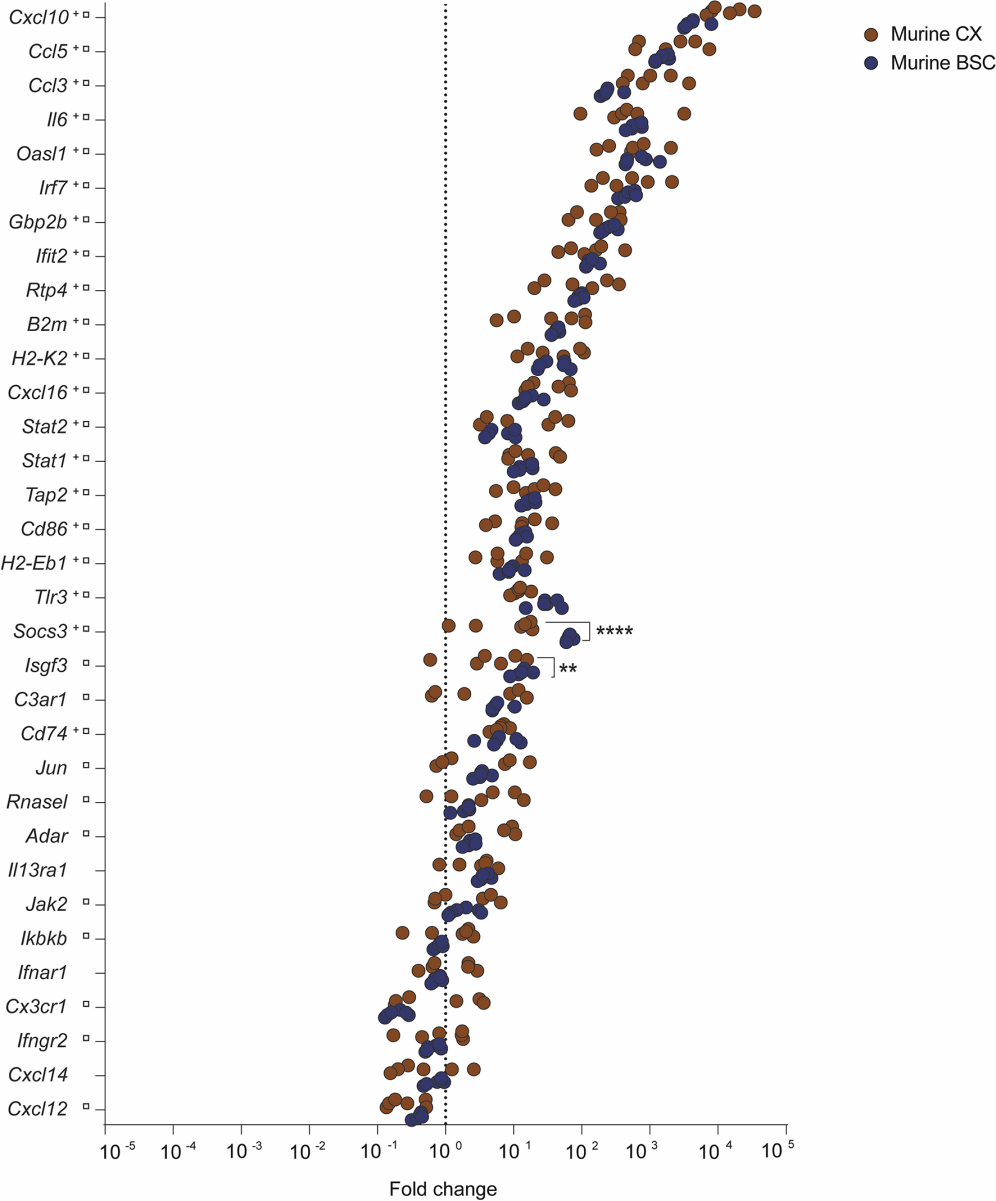
Inflammatory transcription profile of murine CX and BSC upon RABV strain Tha infection at day 8 post-infection. Scatter plot shows gene expression fold changes in murine CX (n=6) and BSC (n=6), upon Tha infection. Fold changes were calculated based on normalization with the geomean of housekeeping genes *Actb* and *Gapdh* (**Supplementary Figure S1**) as well as non-infected murine CX (n=6) and non-infected BSC (n=6), respectively. Differential expression was calculated by comparing the gene expression (ΔΔCT values) between murine CX and BSC with the Šídák’s multiple comparisons test (adjusted p-value ****< 0.0001, **< 0.01). Gene expression in the murine CX (^+^) or murine BSC (**^□^**) that differed significantly from non-infected controls is marked accordingly. A detailed overview of the differential gene expression analysis can be found in **Supplementary Table S4**.

To understand how RABV successfully invades the CNS, we investigated the innate immune gene expression in CX and BSC of mice infected with the cell culture-adapted RABV strain Tha (**Figure 1**). Mice were intramuscularly infected, and RNA was extracted from CX and BSC at day 8 post-infection. At this time point, genes encoding viral nucleoprotein (N-protein), matrix protein (M-protein), and phosphoprotein (P-protein) were present in murine CX and BSC (**Supplementary Figure S2**), confirming RABV infection. Overall, transcription profiles revealed a strong inflammatory response in CX and BSC of Tha-infected mice compared to non-infected animals (**Figure 1** and **Supplementary Figures S3**, **S4**). Further, we defined the number of differentially expressed genes (DEGs) as a significant difference in gene expression (adjusted p-value < 0.05) between infected and non-infected animals (**Supplementary Table S4**). Among the 33 inflammatory genes investigated, we found 20 genes in the murine CX (20 upregulated genes) and 30 genes in the murine BSC (26 upregulated genes, 4 downregulated genes, **Supplementary Table S4**) to be differentially expressed upon Tha infection. In detail, the 20 DEGs observed in the murine CX were also observed in the BSC, presenting a strong universal signature of RABV-induced inflammation in the murine brain (**Supplementary Figure S5**). Similarly, Tha infection strongly induced expression of C-X-C motif chemokine ligand 10 (*Cxcl10*), C-C motif chemokine ligand 5 (*Ccl5*), C-C motif chemokine ligand 3 (*Ccl3*), interleukin 6 (*Il6*), 2’-5’ oligoadenylate synthetase-like 1 (*Oasl1*), interferon regulatory factor (*Irf7*), guanylate binding protein 1 (*Gbp2b*), and interferon induced protein with tetratricopeptide repeats 2 (*Ifit2*) genes in the murine CX and BSC (average fold change > 100). Expression of interferon-stimulated gene factor 3 (*Isfg3*), complement C3a receptor 1 (*C3ar1)*, Jun proto-oncogene (*Jun*), adenosine deaminase RNA specific (*Adar*), ribonuclease L (*Rnasel*) and Janus kinase 2 (*Jak2*) genes was moderately induced in murine CX and BSC (average fold change < 20), but their gene expression was only significantly regulated in the murine BSC. Apart from Tha-induced downregulation of *Cxcl12* and *Cxcl14* in the murine CX and BSC, Tha additionally downregulated expression of nuclear factor kappa B kinase subunit beta (*Ikbkb*), interferon alpha and beta receptor subunit 1 (*Ifnar1*), interferon gamma receptor 2 (*Ifngr2*), *and* C-X3-C motif chemokine receptor 1 (*Cx3cr1*) genes in the murine BSC. When comparing gene expression between the two tissues, *Isfg3* and suppressor of cytokine signalling 3 (*Socs3*) were significantly upregulated in the murine BSC compared to the CX (**Figure 1** and **Supplementary Table S5**).

### The Transcriptome Profile of Natural RABV Infection Revealed Only Limited Inflammatory Responses in the Human BSC Compared to Tha-Infected Murine CX and BSC

**Figure 2:**
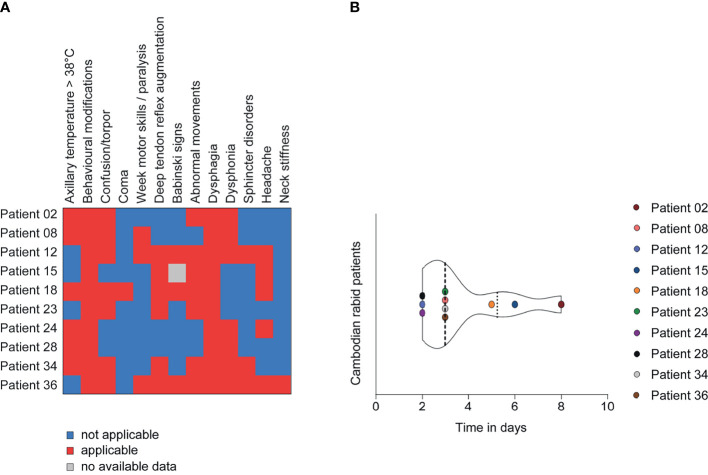
Clinical presentation of Cambodian rabid patients from group 1 (n=10). **(A)** Description of the clinical signs and symptoms of Cambodian rabid patients. **(B)** Time between the onset of symptoms and death in days observed for Cambodian rabid patients. **(A, B)** A detailed overview of the clinical presentation of Cambodian rabid patients belonging to group 1 can be found in **Supplementary Table S1**.

To understand if RABV infection in mice presents a relevant model to study human RABV infection, we compared the results obtained with RABV Tha strain-infected mice to the orthologous gene expression on a cohort of rabid patients from Cambodia (group 1). Group 1 included 10 rabid patients (4 females and 6 males), all infected with canine RABV isolates closely genetically related to the RABV Tha strain used in the mouse model (all RABV isolates belonging to the South-East Asian 3 lineage within the Asian Clade) (**Supplementary Table S1**) (Troupin et al., 2016). These patients were between 8 – 50 years of age (mean= 20.8 years; median= 17 years), and all displayed abnormal behaviour and dysphagia among other symptoms at the time of medical examination (**Figure 2A** and **Supplementary Table S1**). Almost all patients had a history of a rabies exposure after dog bite, ranging back between one year and one month before admission to the hospital. All patients died within 2 – 8 days (mean = 3.7 days, median = 3 days) after the onset of symptoms. The time of brain sample collection corresponds to the time of death (**Figure 2B** and **Supplementary Table S1**). The subset of rabid patients was compared to a subset of 4 non-rabid (control) patients from Cambodia, including 2 females and 2 males, ranging from 41 – 65 years of age (mean= 55 years; median= 57 years) and who died without apparent CNS disorders or infection (**Supplementary Table S1**). Gene expression of viral transcripts was investigated in rabies patients IPC02, IPC08, IPC15, and IPC18, and was significantly different to non-infected controls (patients IPC38, IPC39, IPC40, IPC41, **Supplementary Figure S6**).

**Figure 3:**
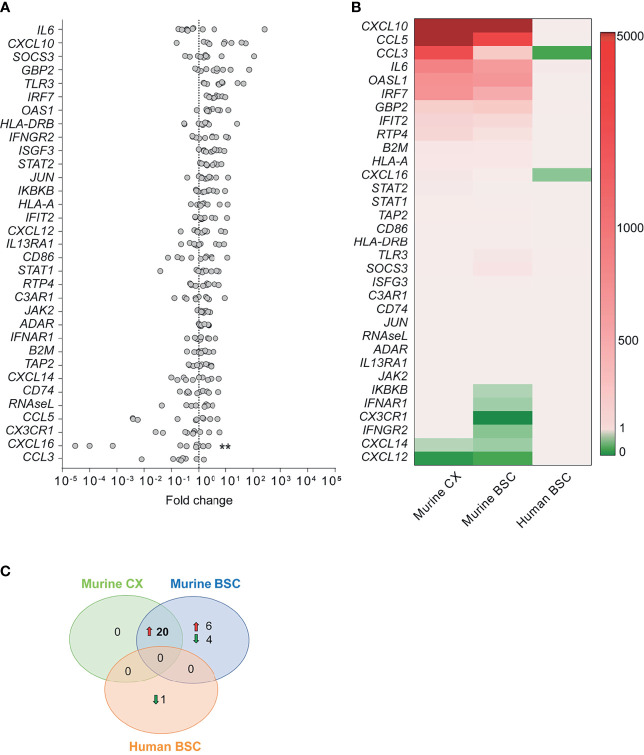
Human transcription profile upon RABV infection. **(A)** Gene expression fold changes in *post-mortem* brain biopsies of the BSC of rabid patients (n=10) normalized to non-rabid patients (n=4). Differential expression was calculated by comparing the gene expression (ΔCT values) between rabid patients (n=10) and non-rabid (control) patients (n=4) by using the Šídák’s multiple comparisons test (** adjusted p-value < 0.01). A detailed overview of the differential gene expression analysis can be found in **Supplementary Table S4**. **(B)** Heatmap comparing the expression of inflammatory genes between RABV Tha strain-infected murine CX (n=6) and BSC (n=6), and RABV-infected human BSC (n=10). Heatmap presents gene expression fold changes calculated from normalized gene expression values (ΔΔCT) of infected animals and humans to the respective non-infected controls (murine CX [n=6]; murine BSC [n=6]; human BSC [n=4]). Gene names are indicated in the human gene name nomenclature although expression indicates quantification of human or murine genes depending on the tissue investigated. **(C)** Number of differentially expressed genes showing the same tendency (up- or downregulation) in the RABV Tha strain-infected murine CX (n=6) or BSC (n=6), and RABV-infected human BSC (n=10). Differential gene expression was calculated to non-infected mice (n=6) or human controls (n=4), respectively. A detailed overview of the differential gene expression analysis can be found in **Supplementary Table S4**.

Using qPCR, we investigated gene expression in the human BSC of rabid patients targeting ortholog inflammatory genes (**Figure 3**) which we previously studied in the murine CX and BSC (**Figure 1**). Unlike the strong innate immune response activation observed in the mouse model upon RABV Tha strain infection (**Figure 1**), the human inflammatory transcriptome profile of natural RABV infection revealed no clear inflammatory response (**Figure 3A**, **Supplementary Table S4** and **Supplementary Figure S7**). Investigating gene expression in naturally infected human brains, only genes coding for IL6, CXCL10, SOCS3, guanylate binding protein 2 (GBP2), and TLR3 showed a modest (average fold change > 10) but not significant upregulation in expression. Further, *CCL3* and *CXCL16* represented the only slightly downregulated genes in the brain of rabid patients compared to non-rabid control patients, with *CXCL16* presenting the only DEG statistically significant in human rabid patients (average fold change = 0.68, **Figures 3A, B** and **Supplementary Table S4**). However, we need to mention that *CXCL16* expression levels varied drastically between rabid patients, with three rabid patients showing a particularly strong reduction in *CXCL16* expression (IPC28, IPC34, and IPC36). Overall, modestly downregulated genes in the human BSC were genes involved in chemotaxis and recruitment of infiltrating leukocytes (*CCL3*, *CXCL16*) suggesting that RABV suppresses recruitment of infiltrating immune cells to the site of infection (**Figures 3A, B**). Similar results were observed in the mouse model, where downregulated genes in the murine CX and BSC are implied in chemotaxis (*Cxcl12*, *Cxcl14*). In contrast, downregulated pathways implied in IFN signalling (*Ifnar1*, *Ifngr2*, *Ikbkb*) in the murine BSC were not observed in the human BSC of rabid patients (**Figure 1** and **Figure 3B**). Apart from *CXCL16* expression, gene expression of other ortholog genes investigated revealed only modest and non-significative modulation in the RABV-infected human BSC (**Figures 3B, C**). Strikingly, and despite the strong similarity of murine CX and BSC transcriptome profiles during RABV Tha strain infection, no gene was found to be differentially up- or downregulated in both, the murine model (murine CX or BSC) and naturally infected rabid patients (**Figure 3C** and **Supplementary Table S4**).

### Regulation of *CXCL16* Expression in the Human and Mouse Brain During RABV Infection

**Figure 4:**
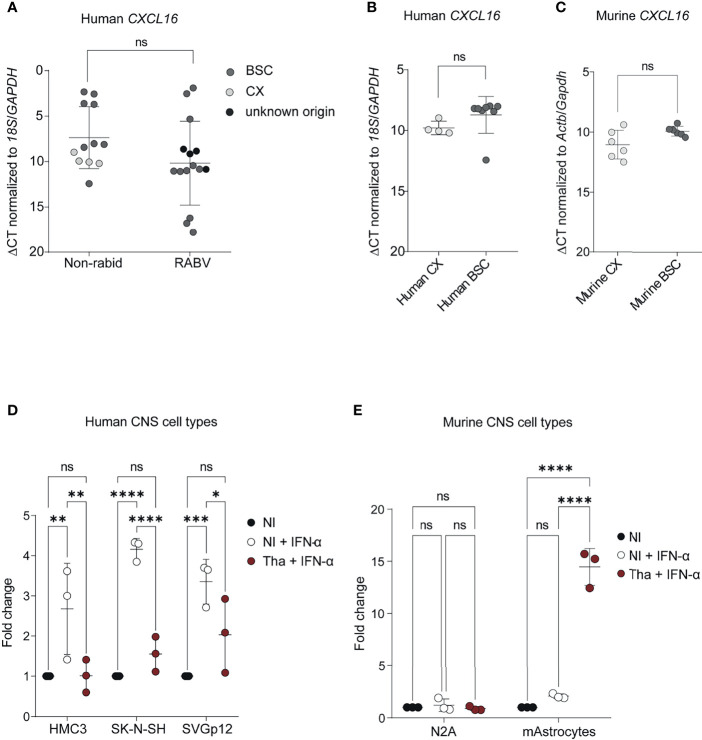
*CXCL16* gene expression in human and murine brains and CNS cell types during RABV infection. **(A)** *CXCL16* gene expression in brain from non-rabid patients and rabid patients (from group 1 and 2). Comparison of *CXCL16* expression in brains from non-rabid (control) patients (n = 12; 12 samples) from Cambodia (IPC38, IPC39, IPC40, IPC41, IPC42) and France (P448, P447, P465, P521, P552, P523, P524) and from rabid patients (n=12; 15 samples) from Cambodia (IPC02, IPC08, IPC12, IPC15, IPC18, IPC23, IPC24, IPC28, IPC34, IPC36), Senegal (IPS01), Moldavia (1900105, 1900106, 1900107), and Madagascar (IPM01). A detailed overview of the additional included human samples can be found in **Supplementary Table S5**. *CXCL16* expression values (ΔCT) were compared by using an unpaired t-test. **(B)** *CXCL16* expression in non-infected control human CX (n=4) and BSC (n=8) samples. *CXCL16* expression values (ΔCT) between CX (P447, P465, P523, P524) and BSC (IPC38, IPC39, IPC40, IPC41, IPC42, P448, P521, P552) were compared by using an unpaired t-test (p=0.072). **(C)** *Cxcl16* expression in the CX (n=6) and BSC (n=6) of non-infected mice. *Cxcl16* expression values (ΔCT) between CX and BSC were compared by using a paired t-test (p=0.122). **(D)** *CXCL16* expression in human CNS cell types (n = 3) infected with RABV Tha (48 hours post-infection). Human CNS cell types (SK-N-SH, HMC3, SVGp12) were infected with Tha (MOI 5) and treated with IFN-α at 24 hours post-infection. **(E)** *Cxcl16* expression in murine neuroblastoma cells and cortical murine astrocytes (n=3) upon RABV Tha strain infection (48 hours post-infection). Murine CNS cell types were infected with Tha (MOI 5) and treated with IFN-α at 24 hours post-infection. Murine astrocytes were extracted from C57BL/6 wild type mice and their phenotype was validated by immunofluorescence and qPCR **(Supplementary Figure S8)**. **(D, E)** All cell culture experiments were performed in three independently replicates. Bars show average fold changed ± SD with a Tukey’s multiple comparison test (adjusted p-value ****< 0.0001, ***< 0.001, **< 0.01, *< 0.05). BSC, brainstem/cerebellum; CX, cortex; HMC3, human microglial cell line; mAstrocytes, cortical murine astrocytes; NI, non-infected; N2A, murine neuroblastoma cells; SK-N-SH, human neuroblastoma cell line; SVGp12, human astroglia cell line; ns, non-significant.

To further investigate the potential downregulation of *CXCL16* by RABV in the human brain, we extended our analysis to an additional group of patients (group 2), which included rabid patients from Moldavia (n=1, 3 samples), Madagascar (n=1, 1 sample) and Senegal (n=1, 1 sample), as well as non-infected control patients from Cambodia (n=1, 1 sample, without apparent CNS disorder or infection) and France (n=4, 7 samples, all presented encephalitis- or meningoencephalitis-related symptoms but without any determined aetiologies) (**Supplementary Table S2**). Thus, this panel included rabid patients from different geographical origins, all being infected with different RABV strains or phylogenetic clades (**Supplementary Table S2**). As presented in **Figure 4A**, we confirmed the tendency of *CXCL16* downregulation during natural RABV infection in the infected human brain (**Figure 4A**) although this downregulation was not found to be significant. To evaluate if the nature of the brain biopsies (i.e. CX or BSC) might be a confounding factor in quantifying *CXCL16* expression observed between infected and control patients, we compared *CXCL16* gene expression between the CX and BSC of non-rabid patients. Based on the results of this comparison, *CXCL16* expression levels were slightly but not significantly higher in the human BSC (n=7) compared to the human CX (n=4), although samples did not originate from the same donors (**Figure 4B**). However, and since most of the human brain samples of rabid patients originated from BSC (n=11) rather than CX (n=4), we can exclude the influence of sampling for the differences of *CXCL16* expression observed in infected RABV patients (**Figure 4B**). At worst, this difference would be minimised, due to the slightly higher expression level of *CXCL16* in the BSC of non-rabid patients (**Figure 4B**). Similarly, *CXCL16* was slightly but not significantly higher expressed in the non-infected murine BSC compared to paired samples taken from the murine CX (**Figure 4C**).

To understand if the difference of *CXCL16* regulation in the murine brain appears due to differences of RABV strains or rather differences in the human versus murine model, we infected different human and murine CNS cell lines with the same RABV Tha isolate previously used for animal infections and evaluated the expression level of *CXCL16* at 48 hours post-infection. For this evaluation, human and murine CNS cell types were infected with Tha (MOI 5) and stimulated at 24 hours post-infection with a hybrid IFN-α which crosses the species barrier to induce *CXCL16* expression. All IFN-α-treated human CNS cell lines, comprising human neuroblastoma cells (SK-N-SH), human microglia-like cells (HMC3), and human astrocyte-like cells (SVGp12), showed a downregulation of *CXCL16* gene expression upon RABV Tha strain infection in contrast to the IFN-α-treated mock (**Figure 4D**). Conversely, *CXCL16* gene expression of murine neuroblastoma N2A cells and cortical astrocytes treated with universal IFN-α remained unchanged (**Figure 4E**). Surprisingly, and in contrast to murine N2A cells, cortical murine astrocytes strongly increased *CXCL16* gene expression upon Tha infection (**Figure 4E**). Thus, activated murine astrocytes might be the source for CXCL16 production in the Tha-infected murine brain.

### RABV Possibly Modifies Neurotransmission and Inflammatory Immune Cell Recruitment in the Human Brain *via* Modulation of *CXCL16* Expression

Lastly, we investigated the modulated pathways in the brain of rabid patients, based on the transcriptomic analysis. First, using the functional enrichment analysis based on gene expression ranks (STRING software), we identified interleukin-10 receptor activity (GO:0004920), C-C chemokine receptor type 5 (CCR5), interferon receptor activity (GO:0004904), CCR1 chemokine receptor binding (GO:0031726), and TAP binding (GO:0046977) as the most upregulated molecular functions found in gene ontology (GO) in human brains which were naturally infected with RABV strains (**Supplementary Figure S9**).

**Figure 5:**
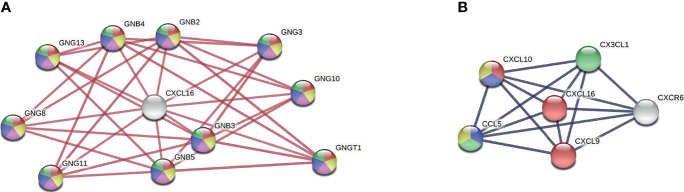
CXCL16 interaction network. **(A)** Physical network of CXCL16 indicating physical protein associations. Colours present five of the most upregulated KEGG pathways (strength > 2, FDR < 5.94e-21): Red = GABAergic synapse (hsa04727), yellow = serotonergic synapse (hsa04726), purple = cholinergic synapse (hsa04725), blue = glutamatergic synapse (hsa04724), green = dopaminergic synapse (hsa04728) **(B)** Full network of CXCL16 indicating functional and physical protein associations. Colours present four of the most upregulated biological processes from gene ontology (strength > 2, FDR < 0.00021): Red = T cell chemotaxis (GO:0010818), blue = regulation of T cell chemotaxis (GO:0010819), green = macrophage chemotaxis (GO:0048246), yellow = positive regulation of monocyte chemotaxis (GO:0090026). Networks were generated by using the STRING version 11.0. GNB, beta unit of guanine nucleotide-binding proteins; GNG, gamma unit of guanine nucleotide-binding proteins.

Secondly, we focused on the only significantly differentially expressed gene, *CXCL16*, during RABV infection, and we modelled physical and full protein interaction networks *via* STRING. The physical interaction network revealed that CXCL16 interacts with the beta and gamma subunits of a range of guanine nucleotide-binding proteins (GNG/GNB) which modulate or transduce various transmembrane signalling systems (**Figure 5A**). Beta and gamma chains are necessary for GTPase activity, to the replacement of GDP by GTP, as well as to the G protein-effector function which mainly conducts the information *via* G-protein-coupled receptors (GPCRs). Most of the GPCR signalling pathways in the brain are involved in synaptic functions: the five most upregulated KEGG pathways of the physical interaction network were involved in signalling pathways of the GABAergic synapse (hsa04727), serotonergic synapse (hsa04726), the cholinergic synapse (hsa04725), the glutamatergic synapse (hsa04724), and the dopaminergic synapse (hsa04728). In short, this analysis suggests that RABV could potentially influence neurotransmission and synaptic plasticity by modulating *CXCL16* gene expression. Apart from physical interaction partners, the full interaction network of *CXCL16* revealed the enrichment of T cell, monocyte, and macrophage chemotactic processes (**Figure 5B**). Consequently, downregulation of *CXCL16* during RABV infection might influence chemotaxis resulting in reduced migration and adhesion of infiltrating immune cells to the infected site.

## Discussion

In this study, we compared for the first time the transcriptome profiles during RABV infection at the brain level in a mouse model and in human cases, focusing on a list of 33 inflammatory genes. The choice of genes was based on a preliminary transcriptome analysis conducted on a limited panel of human (n=4, 2 positive and 2 negative) and murine samples (3 positive and 3 negative) using the Affymetrix microarrays GeneChip^®^ Human Genome U133 Plus 2.0 and GeneChip^®^Mouse Genome 430A 2.0, respectively (data not shown). Based on this selection, the expression of selected genes in the murine CX and BSC was induced upon pathogenic RABV Tha strain infection (canine rabies isolate, cell culture-adapted virus). Further, transcription profiles of murine CX and BSC showed a universal gene signature presenting 20 out of 33 genes which were differentially upregulated in Tha-infected mice compared to non-infected controls (**Supplementary Figure S5**). Between the murine CX and BSC, *Isgf3* and *Socs3* were significantly higher expressed in the murine BSC compared to the CX. Generally, type I and III IFNs induce the formation of transcription factor complex containing Stat2, Stat1, and Irf7, known as Isgf3, which translocates to the nucleus and initiates the transcription of IFN-stimulated genes (ISGs) (Fink and Grandvaux, 2013). In contrast to the induction of ISGs by Isgf3, Socs proteins initiate a negative feedback regulation of the JAK-STAT pathway to ensure the appropriate activation of innate immune pathways (Huang et al., 2020). Higher expression of *Isgf3* and *Socs3* in the murine BSC suggest that RABV strongly activates canonical and non-canonical IFN signaling pathways in the murine BSC resulting in the transcription of ISGs. Further, IFN signaling pathways induce negative feedback regulation pathways *via* Socs proteins. Possible explanations for regional differences in innate immune gene expression within the murine brain may rely on the different capabilities of the CX and BSC in mounting antiviral responses (Fitting et al., 2010; Cho et al., 2013) and/or on the different time spans for viral persistence. Indeed, after im infection, RABV is transported *via* retrograde axonal flux from motor neurons to the CNS (Davis et al., 2015), suggesting that RABV reaches the BSC before reaching the CX. Additionally, viral load in the BSC was slightly higher compared to the murine CX (**Supplementary Figure S2**). Thus, we suggest that the murine BSC mounts a stronger inflammatory response towards virulent RABV Tha strain compared to the murine CX, possibly because of longer viral persistence and the natural capacity of the CX to mount enhanced antiviral responses (Fitting et al., 2010; Cho et al., 2013).

Investigating the expression of inflammatory genes in the brain of rabid patients at the late stage of RABV infection revealed profound differences versus to the mouse model (CX and BSC, **Figure 3**). Compared to the universal gene signature of 20 DEGs observed in the murine brain (CX and BSC), only *CXCL16* (fold change = 0.68) was slightly but significantly downregulated in the BSC of rabid patients from Cambodia (**Figure 3C**). Strikingly, *CXCL16* gene expression presented a large variation among rabid patients, with patients IPC28, IPC34 and IPC36 associated with the highest downregulation (**Figure 3A**). Nowadays, we know that the pathological agent, its infectious load, the host genetic variability, and the surrounding environment strongly shape chemokine and chemokine receptor expression during disease (Flores et al., 2006; Nogueira et al., 2012; Wilson et al., 2013; Qidwai and Khan, 2016). Although the beforementioned parameters might influence the expression of inflammatory genes, we were not able to evaluate these parameters in our study.

Overall, five genes were modestly but not significantly upregulated upon natural RABV infection in humans (*IL6*, *CXCL10*, *SOCS3*, *GBP2*, *TLR3*) whereas two genes were modestly downregulated: *CCL3*, *CXCL16*, the latter being significantly but slightly downregulated. Interestingly, TLR3 was previously shown to be involved in the spatial arrangement of RABV-induced NBs (Ménager et al., 2009) presenting important sites for viral replication (Ni et al., 1996). Although we show only a modest and not significant increase in TLR3 expression in human rabid patients compared to control patients, RABV might exploit TLR3 functions for viral replication as already shown previously (Ménager et al., 2008; Ménager et al., 2009).

Strikingly, *CCL3* was modestly but not significantly downregulated in the BSC of rabid patients from Cambodia (**Figure 3**), whereas *Ccl3* was among the most upregulated genes in the murine brain (**Figure 1**) pointing out profound differences between RABV Tha strain-infected mice and natural RABV infection occurring in humans. *CCL3* bears inflammatory and chemokinetic properties including cellular recruitment and trafficking, immune regulation, and host inflammatory responses (Fitzgerald et al., 2001). Interestingly, it is suggested that *CCL3* plays an important role in macrophage recruitment, T-cell differentiation (Trifilo et al., 2003), and regulation of the BBB permeability (Chui and Dorovini-Zis, 2010). Zhao and colleagues showed that immunizing mice infected with an attenuated RABV with CCL3 at the inoculation site reduced viral pathogenicity and enhanced recruitment of DCs and B cells to the site of immunization (Zhao et al., 2010). Thus, targeted downregulation of *CCL3* by wildtype RABV might possibly restrict BBB opening and recruitment of inflammatory cells, ultimately hindering the clearance of RABV from the infected brain. Nevertheless, further studies are needed to elucidate the function of CCL3 in human rabies.

Although RABV induced overall little modulation of gene expression of innate immunity genes in humans compared to Tha-infected murine brains (**Figure 3** and **Supplementary Figure S7**), we identified CCR5 chemokine receptor binding, CCR1 chemokine receptor binding, interleukin-10 receptor activity, interferon receptor activity, and TAP binding as major signalling pathways implied in the modulation of the investigated genes (**Supplementary Figure S9**). Nevertheless, we need to admit that identification of these pathways was based on modest changes in gene expression levels, which revealed in most of the cases no significant difference to non-infected controls (**Supplementary Table S4**), as well as the prior selection of inflammatory genes investigated. However, and despite these limitations, most of the identified pathways were already described to be involved in RABV infection elsewhere. First, the CCL5/CCR5 axis was already shown to be essential to mediate immune cell infiltration in the CNS (Huang et al., 2014). Besides rabies, CCL5 plays a major role in leukocyte recruitment during herpes virus- and West Nile virus-induced encephalomyelitis (Vilela et al., 2009; Hussmann and Fredericksen, 2014). Recent sequencing data of purified human CNS cells revealed that CCR5 and CCR1 are strongly expressed on infiltrating macrophages and microglia compared to other CNS cell types (Zhang et al., 2014). Further, the transcription factors STAT1 and STAT3 are strongly implicated in the regulation of ligand expression for CCR1/CCR5 signalling. In the past, the interaction between STATs and RABV were well studied, and numerous publications reported modulation of STAT activity by virulent RABV *via* its P-protein (Wiltzer et al., 2012; Lieu et al., 2013; Wiltzer et al., 2014; Sonthonnax et al., 2019). Overall, RABV-mediated modulation of the CCR1/CCR5 axis *via* STAT proteins might hinder macrophage infiltration and microglial activation in the human CNS. Additional modulated pathways imply IFN receptor activity pointing at RABV-modulation of the IFN system. Over the time it has become increasingly clear that the host innate immune system, particularly the IFN system, and RABV-mediated counteractions *via* accessory protein functions profoundly shape the virus-host relationship (Rieder and Conzelmann, 2011). Apart from its STAT interactions, the RABV P-protein of pathogenic RABV strains 1088 and HCM-9 have already been shown to interact with the I-kappa B kinase *in vitro*, thereby inhibiting the induction of IFN signalling and the establishment of an antiviral response (Masatani et al., 2016).

Some differences inherent to the human versus the mouse infection models are associated with our study design, which could influence the results between the two models. First, the Tha RABV strain used in the mouse model (8743THA, GenBank EU293121) is a cell culture-adapted virus which is closely related to the original wildtype strain, collected from a rabid Thai patient (8764THA, GenBank No. EU293111). Despite their close genetic proximity (98.56% identity and 99.36% similarity of the concatenated protein sequences), we cannot exclude that at least one or several of the 52 mutations acquired during the cell culture adaptation could specifically influence the immune response during infection. Similarly, the RABV strain used in the mouse model (originated from Thailand) is phylogenetically closely related to the isolates circulating in Cambodia, all of them belonging to the South East Asian 3 lineage within the Asian clade (Troupin et al., 2016). However, some genetic variations between the viruses ciruclating in Cambodia and the cell culture-adapted Tha could potentially affect the host immune response differently between the two models. Despite these limitations, we provide evidence in our models that human rabies patients show only limited expression of inflammatory genes, whereas the mouse model strongly increased the expression of inflammatory genes (*Cxcl10*, *Ccl5*, *Ccl3*, *Il6*, *Irf7*, *Gbp2b*, *Ifit2*) upon RABV Tha strain infection.

Apart from numerous differences between the human and murine immune system in terms of TLRs, leukocyte subsets, antibody subsets, and defensins (Tao and Reese, 2017), single-nucleus sequencing of human and murine brains have shown marked alterations in cell type proportions and morphologies, laminar distributions, and gene expression (Hodge et al., 2019). Hence, differences in human and murine brains, immune systems and viral adaptation mechanisms (Tao and Reese, 2017; Hodge et al., 2019) as well as differences in viral strains used (natural RABV versus cell adapted RABV) might account for the large difference observed in the establishment of the innate immune response between the RABV-infected human and murine brains.

Further, we provide for the first-time evidence that pathogenic RABV specifically downregulates *CXCL16* expression in the brain of rabid patients, contrasting induction of *CXCL16* expression observed in the murine model. Similarly, RABV Tha strain inhibited IFN-α-mediated induction of *CXCL16* expression in neural, astrocytic, and microglial cell types of human origin but not in murine astrocytes *in vitro*. Although we suggest that activated astrocytes might be the source for *CXCL16* production in the RABV Tha strain-infected murine brain, we cannot exclude that other cells, such as murine microglia, are another important source of CXCL16 upon RABV infection.

CXCL16 is a transmembrane chemokine which can be released in a soluble form from microglia and astrocytes (Ludwig et al., 2005) upon cleavage by metalloproteases ADAM10 and ADAM17 (Trettel et al., 2020). Soluble CXCL16 acts as a chemoattractant for CXCR6/Bonzo-expressing cells such as T cells, NK cells, or glial precursors (Hattermann et al., 2008). Interaction of CXCL16 with CXCR6/Bonzo induces calcium influx, T and NK cell migration and activation *via* the Akt/mammalian target of rapamycin (*mTOR*) pathways (Matloubian et al., 2000; Wang et al., 2008; Günther et al., 2012). Generally, CXCL16 concentrations are increased in inflammatory brain diseases (Ludwig et al., 2005; Le Blanc et al., 2006). In detail, quantification of cortical proteins in a murine model for traumatic brain injury provides evidence of CXCL16 downregulation at all investigated time points. As a consequence, the authors suggest the use of CXCL16 as a potential drug candidate for traumatic brain injuries (Tweedie et al., 2020). Apart from its role in mediating neuro-inflammatory and neurotoxic events in the brain, a role of CXCL16 in mediating crosstalk between astrocytes and neurons has been proposed recently (Rosito et al., 2012; Trettel et al., 2020). Further, CXCL16 induced neuroprotection against excitotoxic damage mediated by excessive glutamate exposure or oxygen glucose deprivation *in vitro* (Rosito et al., 2012). In detail, CXCL16 induces the expression of astrocyte-derived CCL2, which is partly mediating neuroprotection (Rosito et al., 2012). In addition to neuroprotection, CXCL16 exerts a modulatory activity on inhibitory and excitatory synaptic transmission of the murine hippocampus *in vivo* (Di Castro et al., 2016). More specifically, CXCL16 modulates GABA and glutamate release, impacting inhibitory and exhibitory synaptic currents *in vitro* (Di Castro et al., 2016).

Since CXCL16 signaling is implied in neuro-inflammatory as well as neuro-protective and synaptic transmission mechanisms, we can only hypothesize at this stage the effect of RABV-modulated downregulation of *CXCL16* expression in the brain of rabid patients. While chemokines are crucial for the elimination of many different viruses (Nansen et al., 2000; Cook et al., 2004; Alfano and Poli, 2005; Christensen et al., 2006), the viral modulation of chemokine expression can also result in an inappropriate antiviral response and viral persistence in the infected tissue (Melchjorsen et al., 2003), which might be the case in human rabies pathogenesis. We hypothesize that RABV-mediated downregulation of *CXCL16* expression inhibits T cell, NK cell, and glial precursor recruitment and activation enabling RABV to replicate undetected in the human CNS. Although RABV infection seems not to result in excitotoxicity *in vitro* (Weli et al., 2006), CXCL16 might modulate neurotransmission during viral infection. Undoubtedly, investigating the role of *CXCL16* in modulating neuroprotection and synaptic function remains a significant question of interest to better understand how RABV modulates synaptic function.

Overall, species-specific immune responses highlighted the transcriptome differences observed in RABV-infected brains between the mouse model and human cases, thus emphasize the importance of the choice of the most relevant models/samples to study the mechanisms of rabies pathogenesis in humans. This study points out vast differences in the expression of innate immunity genes upon pathogenic RABV infection in mice and in humans: whereas intramuscular infection of mice with a virulent canine RABV results in the upregulation of inflammatory genes in murine CX and BSC, transcriptome analysis of *post-mortem* human brains revealed only little inflammatory responses upon infection with closely related canine RABV isolates. It would be of interest to confirm these results by investigation of the level of expression of the corresponding proteins, which was not possible in our study due to the very limited amount of human samples available. More specifically, our study revealed that RABV specifically downregulates *CXCL16* in the human brain, a mechanism that was not observed in the mouse model. Since *CXCL16* is crucial for the induction of T, NK, and glia cell migration to the infected brain area, we hypothesize that RABV-mediated downregulation of *CXCL16* expression hinders successful viral clearance from the brain. Although animal models have distinct advantages, especially to study RABV pathogenesis, our results emphasize species-specific differences in CXCL16 signaling during RABV infection to show potential limitations of extrapolating transcriptome data from mice to human.

## Data Availability Statement

The original contributions presented in the study are included in the article/**Supplementary Material**. Further inquiries can be directed to the corresponding authors.

## Ethics Statement

The studies involving human participants from Madagascar, Cambodia and Senegal were reviewed and approved by the national ethical committees (N° 102-SANPF/CAB from the Ministry of Health and Family Planning of Madagascar, N°008/03 NECHR and N° 054NECHR from the Ministry of Health of Cambodia, N°00116 MSPM/DS/DER from the Ministry of Health and Medical Prevention of Senegal). Written informed consent to participate to this study was provided by the participants’ legal guardian/next of kin. Samples from the other human participants were included in this study for secondary research use, after informed consent obtained directly from patients or their relatives. The animal study was reviewed and approved by Animal Experimentation Ethics Committee (CETEA no. 089, 25/04/2018 and A75-15-25, 30/07/2002).

## Author Contributions

AH, RR, SD, and CN performed or organized the collection of brain biopsies in human patients in Moldavia, Madagascar, Senegal, and Cambodia, respectively, LF, HB, and LD designed the study; J-MR, PB, and LD supervised the study in Madagascar, Cambodia and in France (overall study), respectively. LF, RL, AL and LD performed the experiments. LF, LD, BR, and IS analysed the data. LF and LD interpreted the data and wrote the manuscript. All authors contributed to the article and approved the submitted version.

## Funding

LF was supported by the Pasteur - Paris University (PPU) International PhD Program. This work was partially funded by Institut Pasteur International Network Actions Concertees InterPasteuriennes (2003/687). We are also grateful to Santé publique France for the financial support of the National Reference Centre for Rabies.

## Conflict of Interest

Author PB is employed by GSK Vaccines.

The remaining authors declare that the research was conducted in the absence of any commercial or financial relationships that could be construed as a potential conflict of interest.

## Publisher’s Note

All claims expressed in this article are solely those of the authors and do not necessarily represent those of their affiliated organizations, or those of the publisher, the editors and the reviewers. Any product that may be evaluated in this article, or claim that may be made by its manufacturer, is not guaranteed or endorsed by the publisher.

## Supplementary Material

Supplementary Figure 1 Validation of human and murine housekeeping genes (*ACTB, GAPDH* and *18S*). **(A)** *Actb* and *Gapdh* presented the lowest pairwise variation for murine CX and BSC and were subsequently selected as murine housekeeping genes to normalize gene expression of murine target genes. **(B)** *18S* and *GAPDH* presented the lowest pairwise variation in human BSC biopsies and were subsequently selected as housekeeping genes to normalize gene expression of human target genes. **(A, B)** Selection of housekeeping genes was performed as described by Vandesompele and colleagues (Vandesompele et al., 2002). *Actb*, actin beta; BSC, brainstem/cerebellum, CX, cortex; *GAPDH*, glyceraldehyde-3-phosphate dehydrogenase.

Supplementary Figure 2 Quantification of RABV N-protein, M-protein, and P-protein gene expression in the murine CX and BSC by qPCR. **(A)** Quantification of viral load in the murine CX by comparing expression of viral genes in Tha-infected (n=3) and non-infected (n=3) mice by specific qPCR (n=6). **(B)** Quantification of viral load in the murine BSC by comparing expression of viral genes in Tha-infected (n=6) and non-infected (n=6) mice by qPCR (n=12). **(A, B)** Differential expressed genes were defined as genes which expression (ΔCT) varied significantly (adjusted p-value < 0.05) between infected samples and non-infected controls by using the Šídák’s multiple comparisons test (**** adjusted p-value < 0.0001). BSC, brain stem/cerebellum; CX, cortex; M-protein, matrix protein; NI, non-infected; N-protein, nucleoprotein; P-protein, phosphoprotein.

Supplementary Figure 3 Clustering of the transcriptome profile of the RABV Tha strain-infected (n=6) and non-infected murine CX (n=6). **(A)** Euclidian clustering of gene expression values revealed a clear separation of two distinct clusters. Euclidian clustering was performed by clustering the gene expression values (ΔCT) using R version 3.4.4. **(B)** PCA of gene expression values revealed a clear separation of two distinct clusters. PCA clustering was performed by clustering the gene expression values (ΔCT) by using R version 3.4.4. **(C)** The determination of the number of clusters was validated by calculating the relative cluster variation. Cluster variation was calculated by dividing the in-cluster variation by the total variation using R version 3.4.4.

Supplementary Figure 4 Clustering of the transcriptome profile of the RABV Tha strain-infected BSC (n=6) and non-infected murine BSC (n=6). **(A)** Euclidian clustering of gene expression values revealed a clear separation of two distinct clusters. Euclidian clustering was performed by clustering the gene expression values (ΔCT) using R version 3.4.4. **(B)** PCA of gene expression values revealed a clear separation of two distinct clusters. PCA clustering was performed by clustering the gene expression values (ΔCT) by using R version 3.4.4. **(C)** The determination of the number of clusters was validated by calculating the relative cluster variation. Cluster variation was calculated by dividing the in-cluster variation by the total variation using R version 3.4.4.

Supplementary Figure 5 Pathways activated in the murine CX and BSC. Universal signalling pathways underlying modulation of innate immunity gene expression in murine CX and BSC upon RABV strain Tha infection. Colours present the five most presented molecular functions from gene ontology (strength > 2.02, FDR < 0.00035): red = CCR5 chemokine receptor binding (GO:0031730); blue = phospholipase activator activity (GO:0016004); green = chemokine activity (GO:008009); yellow = chemokine receptor binding (GO:0042379); purple = CCR chemokine receptor binding (GO:0048020). Networks were generated by performing a functional enrichment analysis based on gene expression ranks and SD of the universal gene signature in murine CX and BSC (expression of 20 DEGs) using the STRING version 11.0.

Supplementary Figure 6 Quantification of RABV N-protein, M-protein, and P-protein gene expression in the human BSC by qPCR. In detail, expression of viral genes in non-infected human controls (n=4; IPC38, IPC39, IPC40, IPC41) and rabid patients (n=4; IPC02, IPC08, IPC12, IPC15) were quantified in the human BSC by qPCR (n=8). Differential expressed genes were defined as genes which expression (ΔCT) varied significantly (adjusted p-value < 0.05) between infected samples and non-infected controls by using the Šídák’s multiple comparisons test (*** adjusted p-value < 0.001, * adjusted p-value < 0.05). BSC, brain stem/cerebellum; M-protein, matrix protein; NI, non-infected; N-protein, nucleoprotein; P-protein, phosphoprotein.

Supplementary Figure 7 Clustering of the transcriptome profile of the RABV-infected (n=10) and non-infected (control) (n=4) human post-mortem brain biopsies from group 1. **(A)**. Euclidian clustering of gene expression values revealed three distinct clusters. Euclidian clustering was performed by clustering the gene expression values (ΔCT) using R version 3.4.4. **(B)** PCA of gene expression values revealed a separation into three distinct clusters. PCA clustering was performed by clustering the gene expression values (ΔCT) by using R version 3.4.4. **(C)** The determination of the number of clusters was validated by calculating the relative cluster variation. Cluster variation was calculated by dividing the in-cluster variation by the total variation using R version 3.4.4.

Supplementary Figure 8 Validation of astrocytic phenotype. **(A)** Representative images showing GFAP staining of mAstrocytes **(B)** Quantification of relative *Gfap* expression of murine N2A cells and mAstrocytes isolated from murine pups. All experiments were performed three times independently. Bars show the mean ΔCT values ± SD. Differential expression was calculated by applying an unpaired t-test (**** p-value < 0.0001). GFAP, Glial fibrillary acidic protein; mAstrocytes, murine cortical astrocytes; NI, non-infected; N-protein, nucleoprotein.

Supplementary Figure 9 Pathways activated in the post-mortem brain of rabid patients. Colours present the five most presented molecular functions from gene ontology (strength > 2.27, FDR < 0.0011): red = interleukin 10 receptor activity (GO:0004920), blue = CCR5 chemokine receptor binding (GO:0031730); green = interferon receptor activity (GO:0004904), yellow = CCR1 chemokine receptor binding (GO:0031726), purple = TAP binding (GO:0046977). Networks were generated by performing a functional enrichment analysis based on gene expression ranks and SD using the STRING version 11.0.

## Glossary

**Table:**

*Actb*	actin beta
BSC	brain stem/cerebellum
CD	cluster of differentiation
cDNA	complementary DNA
CNS	central nervous system
*Ccl*	CC-chemokine ligand
CCR	C-C chemokine receptor
CX	cortex
*Cxcl*	C-X-C motif chemokine ligand
*Cxcr*	C-X-C motif chemokine receptor
DEGs	differentially expressed genes
*GAPDH*	glyceraldehyde-3-phosphate dehydrogenase
G protein	glycoprotein
GNB	beta unit of guanine nucleotide-binding proteins
GNG	gamma unit of guanine nucleotide-binding proteins
ic	intracerebral
IFN	interferon
*Ifnar*	interferon-α/β receptor
*Ifngr2*	interferon gamma receptor 2
*Ikbkb*	inhibitor of nuclear factor kappa B kinase subunit beta
IL	interleukin
im	intramuscular
ISG	interferon stimulated genes
*Isgf3*	*interferon stimulated gene factor 3*
*Jak*	*Janus kinase*
*mAstrocytes*	murine cortical astrocytes
M-protein	matrix protein
MOI	multiplicity of infection
NBs	Negri bodies
NK cells	natural killer cells
N2a	Neuro-2a cells
N-protein	nucleoprotein
*Oasl1*	*5’-oligoadenylate synthetase-like protein 1*
PNS	peripheral nervous system
P-protein	phosphoprotein
qPCR	quantitative PCR
RABV	rabies virus
SD	standard deviation
*Stat*	signal transducer and activator of transcription 1
TLRs	toll-like receptors
wt	wildtype
