# Supplementary material for: Transcriptome Profile During Rabies Virus Infection: Identification of Human CXCL16 as a Potential New Viral Target
Source: Front Cell Infect Microbiol. 2021 Nov 5;11:761074. doi: 10.3389/fcimb.2021.761074 (PMC8602097; doi:10.3389/fcimb.2021.761074)
Supplement: Supplementary Figure 1 — Validation of human and murine housekeeping genes (ACTB, GAPDH and 18S). (A) Actb and Gapdh presented the lowest pairwise variation for murine CX and BSC and were subsequently selected as murine housekeeping genes to normalize gene expression of murine target genes. (B) 18S and GAPDH presented the lowest pairwise variation in human BSC biopsies and were subsequently selected as housekeeping genes to normalize gene expression of human target genes. (A, B) Selection of housekeeping genes was performed as described by Vandesompele and colleagues (Vandesompele et al., 2002). Actb, actin beta; BSC, brainstem/cerebellum, CX, cortex; GAPDH, glyceraldehyde-3-phosphate dehydrogenase. [file Presentation_1.zip › Supplementary Material_updated/Table_S1.docx]

**Table S1. Epidemiological and clinical data of the first group of patients included in this study (rabid (n=10) and control (n=4) patients from Cambodia).**

| **Patient ID** | **Sample ID** | **Sex (M = male F = female)** | **Age range (year)** | **Infection status** | **Bite history (dog)** | **Time between onset of symptoms and hospitalization (day)** | **Time from onset of symptoms until death (day)** | **Axillary temperature at admission** | **Behavioural disorders** | **Confusion/ torpor** | **Coma** |
| --- | --- | --- | --- | --- | --- | --- | --- | --- | --- | --- | --- |
| 02 | IPC02 | F | 10-15 | Positive | Yes | 3 | 8 | ≥ 38°C | Yes | Yes | No |
| 08 | IPC08 | F | 5-10 | Positive | Yes | 1 | 3 | ≥ 38°C | Yes | Yes | No |
| 12 | IPC12 | M | 25-30 | Positive | Yes | 1 | 2 | < 38°C | Yes | Yes | No |
| 15 | IPC15 | M | 20-25 | Positive | NA | 5 | 6 | < 38°C | Yes | No | No |
| 18 | IPC18 | M | 5-10 | Positive | Yes | 4 | 5 | ≥ 38°C | Yes | Yes | Yes |
| 23 | IPC23 | F | 10-15 | Positive | Yes | 2 | 3 | < 38°C | Yes | Yes | No |
| 24 | IPC24 | F | 30-35 | Positive | Yes | 1 | 2 | ≥ 38°C | Yes | No | No |
| 28 | IPC28 | M | 5-10 | Positive | Yes | 1 | 2 | ≥ 38°C | Yes | No | No |
| 34 | IPC34 | M | 50-55 | Positive | Yes | 3 | 3 | ≥ 38°C | Yes | Yes | No |
| 36 | IPC36 | M | 30-35 | Positive | Yes | 2 | 3 | < 38°C | Yes | Yes | No |
| 38^a^ | IPC38 | M | 65-70 | Negative | - | - | - | - | - | - | - |
| 39^a^ | IPC39 | F | 55-60 | Negative | - | - | - | - | - | - | - |
| 40^a^ | IPC40 | M | 55-60 | Negative | - | - | - | - | - | - | - |
| 42^a^ | IPC42 | F | 40-45 | Negative | - | - | - | - | - | - | - |

NA: Not applicable; a: These patients did not present apparent CNS disorders or infection.

**Table S1. Epidemiological and clinical data of the group 1 of patients included in this study (rabid (n=10) and control (n=4) patients from Cambodia) (continued)**

| **Patient ID** | **Sample ID** | **Weak motor skills/ paralysis** | **Deep tendon reflex augmentation** | **Babinski signs** | **Abnormal movements** | **Dysphagia** | **Dysphonia** | **Sphincter disorders** | **Neck stiffness** | **Turbid CSF** | **Malaria positive** |
| --- | --- | --- | --- | --- | --- | --- | --- | --- | --- | --- | --- |
| 02 | IPC02 | No | No | No | Yes | Yes | Yes | No | No | Yes | Yes |
| 08 | IPC08 | No | No | No | No | Yes | Yes | No | No | NA | NA |
| 12 | IPC12 | Yes | Yes | Yes | Yes | Yes | No | Yes | No | No | No |
| 15 | IPC15 | No | Yes | - | Yes | Yes | No | No | No | NA | No |
| 18 | IPC18 | No | Yes | Yes | Yes | Yes | No | No | No | NA | NA |
| 23 | IPC23 | No | Yes | No | Yes | Yes | Yes | No | No | NA | No |
| 24 | IPC24 | No | No | No | No | Yes | Yes | No | No | NA | No |
| 28 | IPC28 | No | No | No | No | Yes | Yes | No | No | NA | No |
| 34 | IPC34 | No | Yes | No | Yes | Yes | Yes | Yes | No | No | Yes |
| 36 | IPC36 | Yes | Yes | Yes | Yes | Yes | Yes | Yes | Yes | No | No |
| 38^a^ | IPC38 | - | - | - | - | - | - | - | - | - | - |
| 39^a^ | IPC39 | - | - | - | - | - | - | - | - | - | - |
| 40^a^ | IPC40 | - | - | - | - | - | - | - | - | - | - |
| 42^a^ | IPC42 | - | - | - | - | - | - | - | - | - | - |

NA: Not applicable; a: These patients did not present apparent CNS disorders or infection.
