# Supplementary material for: Transcriptome Profile During Rabies Virus Infection: Identification of Human CXCL16 as a Potential New Viral Target
Source: Front Cell Infect Microbiol. 2021 Nov 5;11:761074. doi: 10.3389/fcimb.2021.761074 (PMC8602097; doi:10.3389/fcimb.2021.761074)
Supplement: Supplementary Figure 1 — Validation of human and murine housekeeping genes (ACTB, GAPDH and 18S). (A) Actb and Gapdh presented the lowest pairwise variation for murine CX and BSC and were subsequently selected as murine housekeeping genes to normalize gene expression of murine target genes. (B) 18S and GAPDH presented the lowest pairwise variation in human BSC biopsies and were subsequently selected as housekeeping genes to normalize gene expression of human target genes. (A, B) Selection of housekeeping genes was performed as described by Vandesompele and colleagues (Vandesompele et al., 2002). Actb, actin beta; BSC, brainstem/cerebellum, CX, cortex; GAPDH, glyceraldehyde-3-phosphate dehydrogenase. [file Presentation_1.zip › Supplementary Material_updated/Table_S2.docx]

**Table S2. Epidemiological and clinical data of the group 2 of patients included in this study.** This second group encompasses rabid patients from Moldavia (n=1, 3 samples), Madagascar (n=1, 1 sample), and Senegal (n=1, 1 sample), as well as control patients from Cambodia (n=1, 1 sample) and France (n=7, 7 samples).

| **Patient ID** | **Sample ID** | **Sex** | **Age range**  **(year)** | **Country** | **Infection status** | **RABV clade**  **(Animal reservoir)^a^** | **Type of biopsy** | **Year of sample collection** |
| --- | --- | --- | --- | --- | --- | --- | --- | --- |
| 43 | 1900105 | M | 10-15 | Moldavia | Positive | Cosmopolitan (red fox/ raccoon dog) | Cerebellum | 2019 |
| 43 | 1900106 | M | 10-15 | Moldavia | Positive | Cosmopolitan (red fox/ raccoon dog) | Hippocampus | 2019 |
| 43 | 1900107 | M | 10-15 | Moldavia | Positive | Cosmopolitan (red fox/ raccoon dog) | Brain stem | 2019 |
| 51^b^ | IPC41 | F | 20-25 | Cambodia | Negative | - | Brain stem/ cerebellum | 2006 |
| 52^c^ | P447 | F | 25-30 | France | Negative | - | Cortex | 2007 |
| 52^c^ | P448 | F | 25-30 | France | Negative | - | Cerebellum | 2007 |
| 53^c^ | P465 | M | 15-20 | France | Negative | - | Cortex | 2003 |
| 54^c^ | P521 | M | 20-25 | France | Negative | - | Cerebellum | 2008 |
| 55^c^ | P522 | F | 30-35 | France | Negative | - | Cerebellum | 2008 |
| 55^c^ | P523 | F | 30-35 | France | Negative | - | Right frontal cortex | 2008 |
| 55^c^ | P524 | F | 30-35 | France | Negative | - | Right occipital cortex | 2008 |
| 56 | IPS01 (07023) | M | 10-15 | Senegal | Positive | Africa 2 (dog) | Brain stem/ cerebellum | 2005 |
| 57 | IPM01 | F | 20-25 | Madagascar | Positive | Cosmopolitan (dog) | Brain stem/cerebellum | 2004 |

a: According to Troupin *et al*., 2016 (Troupin et al., 2016); b: This patient did not present an apparent CNS disorder or infection; c: These patients presented encephalitis- or meningoencephalitis-related symptoms but without any determined aetiologies.
