# Supplementary material for: Transcriptome Profile During Rabies Virus Infection: Identification of Human CXCL16 as a Potential New Viral Target
Source: Front Cell Infect Microbiol. 2021 Nov 5;11:761074. doi: 10.3389/fcimb.2021.761074 (PMC8602097; doi:10.3389/fcimb.2021.761074)
Supplement: Supplementary Figure 1 — Validation of human and murine housekeeping genes (ACTB, GAPDH and 18S). (A) Actb and Gapdh presented the lowest pairwise variation for murine CX and BSC and were subsequently selected as murine housekeeping genes to normalize gene expression of murine target genes. (B) 18S and GAPDH presented the lowest pairwise variation in human BSC biopsies and were subsequently selected as housekeeping genes to normalize gene expression of human target genes. (A, B) Selection of housekeeping genes was performed as described by Vandesompele and colleagues (Vandesompele et al., 2002). Actb, actin beta; BSC, brainstem/cerebellum, CX, cortex; GAPDH, glyceraldehyde-3-phosphate dehydrogenase. [file Presentation_1.zip › Supplementary Material_updated/Table_S4.docx]

**Table S4.** **Identification of** **differentially expressed genes in the murine CX (n = 6), the murine BSC (n=6), and the human BSC (n = 10) upon RABV infection.** Differential gene expression was calculated by comparing the gene expression values (ΔCT) of infected tissues (murine CX [n=6]; murine BSC [n=6]; murine BSC [n=6]) against gene expression values (ΔCT) non-infected tissues (murine CX [n=6]; murine BSC [n=6]; human BSC [n=4]). Differential expressed genes were defined as genes which expression (ΔCT) varied significantly (adjusted p-value < 0.05) between infected samples and non-infected controls by using the Šídák's multiple comparisons test (**** p-value < 0.0001, *** p-value < 0.001 ** p-value <0.01, * p-value < 0.05). CX = cortex; BSC = brain stem/cerebellum; FC = fold change; SD = standard deviation

| **Tissue** | **FC** | **SD** | **Gene** | **Mean Diff.** | **95.00% CI of diff.** | **Below threshold** | **Summary** | **Adjusted P Value** |
| --- | --- | --- | --- | --- | --- | --- | --- | --- |
| Murine CX | 5,41043333 | 4,17336464 | *Adar* | 1,97 | -0,5271 to 4,467 | No | ns | 0,3351 |
| Murine CX | 57,6752333 | 48,1345822 | *B2m* | 5,122 | 2,626 to 7,619 | Yes | **** | <0,0001 |
| Murine CX | 6,5699 | 6,40830455 | *C3ar1* | 1,732 | -0,7644 to 4,229 | No | ns | 0,6015 |
| Murine CX | 1407,53233 | 1290,70618 | *Ccl3* | 9,996 | 7,500 to 12,49 | Yes | **** | <0,0001 |
| Murine CX | 2976,48505 | 2649,82859 | *Ccl5* | 10,98 | 8,486 to 13,48 | Yes | **** | <0,0001 |
| Murine CX | 6,47377034 | 1,48127516 | *Cd74* | 5,695 | 3,198 to 8,191 | Yes | **** | <0,0001 |
| Murine CX | 15,4055522 | 11,9730559 | *Cd86* | 3,969 | 1,472 to 6,466 | Yes | **** | <0,0001 |
| Murine CX | 1,47781667 | 1,55966182 | *Cx3cr1* | -0,4415 | -2,938 to 2,055 | No | ns | >0,9999 |
| Murine CX | 15606,4453 | 10587,781 | *Cxcl10* | 13,68 | 11,18 to 16,18 | Yes | **** | <0,0001 |
| Murine CX | 0,29458333 | 0,17699206 | *Cxcl12* | -1,982 | -4,479 to 0,5149 | No | ns | 0,3234 |
| Murine CX | 0,8222 | 0,95224898 | *Cxcl14* | -1,041 | -3,538 to 1,456 | No | ns | 0,9988 |
| Murine CX | 38,307 | 24,9394863 | *Cxcl16* | 4,97 | 2,473 to 7,467 | Yes | **** | <0,0001 |
| Murine CX | 217,83585 | 134,409958 | *Gbp2b* | 7,47 | 4,973 to 9,967 | Yes | **** | <0,0001 |
| Murine CX | 12,2421167 | 10,2723461 | *H2eb1* | 3,183 | 0,6865 to 5,680 | Yes | ** | 0,002 |
| Murine CX | 51,44265 | 40,9869805 | *H2k2* | 5,213 | 2,716 to 7,710 | Yes | **** | <0,0001 |
| Murine CX | 168,3724 | 140,583771 | *Ifit2* | 7,011 | 4,514 to 9,508 | Yes | **** | <0,0001 |
| Murine CX | 1,4903 | 1,04376387 | *Ifnar1* | 0,2079 | -2,289 to 2,705 | No | ns | >0,9999 |
| Murine CX | 1,11641667 | 0,73058391 | *Ifngr2* | -0,2623 | -2,759 to 2,235 | No | ns | >0,9999 |
| Murine CX | 1,56068333 | 0,92200363 | *Ikbkb* | 0,2541 | -2,243 to 2,751 | No | ns | >0,9999 |
| Murine CX | 3,24408333 | 1,82930531 | *IL13ra1* | 1,433 | -1,064 to 3,930 | No | ns | 0,9018 |
| Murine CX | 846,753082 | 1161,86195 | *Il6* | 8,87 | 6,374 to 11,37 | Yes | **** | <0,0001 |
| Murine CX | 706,474617 | 740,886214 | *Irf7* | 8,849 | 6,352 to 11,35 | Yes | **** | <0,0001 |
| Murine CX | 6,65520068 | 5,57083351 | *Isgf3* | 2,125 | -0,3719 to 4,622 | No | ns | 0,2058 |
| Murine CX | 2,83211667 | 2,4238016 | *Jak2* | 0,9431 | -1,554 to 3,440 | No | ns | 0,9998 |
| Murine CX | 6,04025 | 6,52195467 | *Jun* | 1,633 | -0,8638 to 4,130 | No | ns | 0,7181 |
| Murine CX | 724,163183 | 682,864103 | *Oasl1* | 9,025 | 6,529 to 11,52 | Yes | **** | <0,0001 |
| Murine CX | 5,73679397 | 5,38505415 | *Rnasel* | 1,76 | -0,7368 to 4,257 | No | ns | 0,5684 |
| Murine CX | 141,4559 | 130,371641 | *Rtp4* | 6,335 | 3,838 to 8,831 | Yes | **** | <0,0001 |
| Murine CX | 11,278394 | 7,55865673 | *Socs3* | 2,923 | 0,4259 to 5,420 | Yes | ** | 0,0073 |
| Murine CX | 22,1523833 | 17,7313405 | *Stat1* | 4,089 | 1,592 to 6,586 | Yes | **** | <0,0001 |
| Murine CX | 25,25035 | 24,3605225 | *Stat2* | 3,838 | 1,342 to 6,335 | Yes | **** | <0,0001 |
| Murine CX | 19,78825 | 12,7925832 | *Tap2* | 4,023 | 1,527 to 6,520 | Yes | **** | <0,0001 |
| Murine CX | 12,1701167 | 3,13923435 | *Tlr3* | 3,569 | 1,073 to 6,066 | Yes | *** | 0,0002 |
| Murine BSC | 2,31658417 | 0,36957573 | *Adar* | 1,196 | 0,3857 to 2,007 | Yes | *** | 0,0001 |
| Murine BSC | 41,8437983 | 4,21294634 | *B2m* | 5,381 | 4,570 to 6,191 | Yes | **** | <0,0001 |
| Murine BSC | 6,08920833 | 2,10754222 | *C3ar1* | 2,55 | 1,740 to 3,361 | Yes | **** | <0,0001 |
| Murine BSC | 253,389 | 82,5658044 | *Ccl3* | 7,934 | 7,124 to 8,745 | Yes | **** | <0,0001 |
| Murine BSC | 1529,84033 | 304,187334 | *Ccl5* | 10,56 | 9,745 to 11,37 | Yes | **** | <0,0001 |
| Murine BSC | 7,18888093 | 3,82580426 | *Cd74* | 2,662 | 1,852 to 3,473 | Yes | **** | <0,0001 |
| Murine BSC | 12,913725 | 1,96144682 | *Cd86* | 3,677 | 2,867 to 4,488 | Yes | **** | <0,0001 |
| Murine BSC | 0,19884583 | 0,06674852 | *Cx3cr1* | -2,399 | -3,209 to -1,588 | Yes | **** | <0,0001 |
| Murine BSC | 4428,51417 | 1782,97081 | *Cxcl10* | 12,04 | 11,23 to 12,85 | Yes | **** | <0,0001 |
| Murine BSC | 0,4054955 | 0,0489468 | *Cxcl12* | -1,312 | -2,122 to -0,5013 | Yes | **** | <0,0001 |
| Murine BSC | 0,736865 | 0,19520895 | *Cxcl14* | -0,4878 | -1,298 to 0,3227 | No | ns | 0,8487 |
| Murine BSC | 16,7667583 | 5,68180813 | *Cxcl16* | 4,009 | 3,199 to 4,820 | Yes | **** | <0,0001 |
| Murine BSC | 253,512483 | 57,3089575 | *Gbp2b* | 7,955 | 7,145 to 8,766 | Yes | **** | <0,0001 |
| Murine BSC | 9,394062 | 2,771985 | *H2eb1* | 3,184 | 2,374 to 3,995 | Yes | **** | <0,0001 |
| Murine BSC | 42,4822967 | 19,3908271 | *H2k2* | 5,274 | 4,463 to 6,085 | Yes | **** | <0,0001 |
| Murine BSC | 135,074317 | 25,9787598 | *Ifit2* | 7,058 | 6,247 to 7,869 | Yes | **** | <0,0001 |
| Murine BSC | 0,7519725 | 0,11009312 | *Ifnar1* | -0,4243 | -1,235 to 0,3862 | No | ns | 0,9639 |
| Murine BSC | 0,6632765 | 0,15320989 | *Ifngr2* | -3,611 | -4,421 to -2,800 | Yes | **** | <0,0001 |
| Murine BSC | 0,79779867 | 0,09527985 | *Ikbkb* | 1,72 | 0,9090 to 2,530 | Yes | **** | <0,0001 |
| Murine BSC | 3,69090333 | 0,65131545 | *IL13ra1* | 0,5374 | -0,2731 to 1,348 | No | ns | 0,6925 |
| Murine BSC | 616,74735 | 127,569327 | *Il6* | 9,604 | 8,794 to 10,41 | Yes | **** | <0,0001 |
| Murine BSC | 487,0728 | 107,839538 | *Irf7* | 1,362 | 0,5519 to 2,173 | Yes | **** | <0,0001 |
| Murine BSC | 13,4616861 | 3,44334135 | *Isgf3* | 3,712 | 2,901 to 4,522 | Yes | **** | <0,0001 |
| Murine BSC | 2,0229775 | 0,96465063 | *Jak2* | 3,326 | 2,516 to 4,137 | Yes | **** | <0,0001 |
| Murine BSC | 3,421986 | 0,74168175 | *Jun* | 1,527 | 0,7163 to 2,337 | Yes | **** | <0,0001 |
| Murine BSC | 730,388043 | 375,147309 | *Oasl1* | 9,373 | 8,562 to 10,18 | Yes | **** | <0,0001 |
| Murine BSC | 1,9564675 | 0,40935712 | *Rnasel* | 7,719 | 6,909 to 8,530 | Yes | **** | <0,0001 |
| Murine BSC | 92,45179 | 10,0344433 | *Rtp4* | 6,523 | 5,713 to 7,334 | Yes | **** | <0,0001 |
| Murine BSC | 64,9956617 | 5,88793874 | *Socs3* | 5,42 | 4,610 to 6,231 | Yes | **** | <0,0001 |
| Murine BSC | 14,7167012 | 3,77013853 | *Stat1* | 4,566 | 3,755 to 5,377 | Yes | **** | <0,0001 |
| Murine BSC | 7,01370267 | 3,14478393 | *Stat2* | 5,021 | 4,211 to 5,832 | Yes | **** | <0,0001 |
| Murine BSC | 17,160085 | 3,07182457 | *Tap2* | 1,231 | 0,4206 to 2,042 | Yes | **** | <0,0001 |
| Murine BSC | 32,720055 | 12,7085994 | *Tlr3* | 6,79 | 5,979 to 7,600 | Yes | **** | <0,0001 |
| Human BSC | 1,49963 | 0,54087829 | *ADAR* | 0,5059 | -3,291 to 4,303 | No | ns | >0,9999 |
| Human BSC | 1,48377 | 0,92003872 | *B2M* | 0,3182 | -3,479 to 4,115 | No | ns | >0,9999 |
| Human BSC | 1,87813 | 2,72767458 | *C3AR1* | -0,06979 | -3,867 to 3,727 | No | ns | >0,9999 |
| Human BSC | 0,39254 | 0,45163512 | *CCL3* | -2,212 | -6,009 to 1,584 | No | ns | 0,8875 |
| Human BSC | 1,22515 | 1,58441793 | *CCL5* | -2,199 | -5,996 to 1,597 | No | ns | 0,8936 |
| Human BSC | 1,4415 | 1,54104723 | *CD74* | -0,127 | -3,924 to 3,670 | No | ns | >0,9999 |
| Human BSC | 2,33129 | 3,42360064 | *CD86* | -0,07576 | -3,873 to 3,721 | No | ns | >0,9999 |
| Human BSC | 1,21757 | 1,81675719 | *CXC3R1* | -1,068 | -4,865 to 2,729 | No | ns | >0,9999 |
| Human BSC | 13,52589 | 18,3479195 | *CXCL10* | 2,277 | -1,520 to 6,073 | No | ns | 0,8545 |
| Human BSC | 2,5388 | 3,07608824 | *CXCL12* | 0,5531 | -3,244 to 4,350 | No | ns | >0,9999 |
| Human BSC | 1,44229 | 1,72003307 | *CXCL14* | -0,459 | -4,256 to 3,338 | No | ns | >0,9999 |
| Human BSC | 0,68257 | 0,73182172 | *CXCL16* | -4,437 | -8,233 to -0,6398 | Yes | ** | 0,0074 |
| Human BSC | 11,36797 | 22,020487 | *GBP2* | 1,995 | -1,802 to 5,792 | No | ns | 0,9626 |
| Human BSC | 2,75966 | 3,57710324 | *HLA-A* | 0,7508 | -3,046 to 4,548 | No | ns | >0,9999 |
| Human BSC | 3,86069 | 7,87893681 | *HLA-DRB* | 0,6059 | -3,191 to 4,403 | No | ns | >0,9999 |
| Human BSC | 2,6687 | 3,21755471 | *IFIT2* | 0,8374 | -2,959 to 4,634 | No | ns | >0,9999 |
| Human BSC | 1,49497 | 1,1089224 | *IFNAR1* | 0,2728 | -3,524 to 4,070 | No | ns | >0,9999 |
| Human BSC | 3,80059 | 4,19251398 | *IFNGR2* | 1,133 | -2,664 to 4,929 | No | ns | >0,9999 |
| Human BSC | 2,77401 | 2,70402784 | *IKBKB* | 0,9967 | -2,800 to 4,793 | No | ns | >0,9999 |
| Human BSC | 2,38862 | 2,90426615 | *IL13RA1* | 0,3402 | -3,457 to 4,137 | No | ns | >0,9999 |
| Human BSC | 27,48837 | 84,1645508 | *IL6* | 0,01551 | -3,781 to 3,812 | No | ns | >0,9999 |
| Human BSC | 4,50255 | 2,50721876 | *IRF7* | 1,987 | -1,810 to 5,783 | No | ns | 0,9645 |
| Human BSC | 3,56378 | 2,47803213 | *ISFG3* | 1,528 | -2,268 to 5,325 | No | ns | 0,9994 |
| Human BSC | 1,61002 | 0,50959908 | *JAK2* | 0,6218 | -3,175 to 4,419 | No | ns | >0,9999 |
| Human BSC | 2,96283 | 3,48524733 | *JUN* | 0,9568 | -2,840 to 4,754 | No | ns | >0,9999 |
| Human BSC | 3,86082 | 3,48443708 | *OAS1L* | 1,542 | -2,255 to 5,339 | No | ns | 0,9993 |
| Human BSC | 1,41476 | 0,98149199 | *RNAseL* | -0,1246 | -3,921 to 3,672 | No | ns | >0,9999 |
| Human BSC | 1,93088 | 1,65112514 | *RTP4* | 0,4709 | -3,326 to 4,268 | No | ns | >0,9999 |
| Human BSC | 12,65473 | 34,0692787 | *SOCS3* | 1,017 | -2,780 to 4,814 | No | ns | >0,9999 |
| Human BSC | 2,06999 | 1,76849991 | *STAT1* | 0,3241 | -3,473 to 4,121 | No | ns | >0,9999 |
| Human BSC | 3,10008 | 1,88016111 | *STAT2* | 1,394 | -2,402 to 5,191 | No | ns | 0,9999 |
| Human BSC | 1,44704 | 0,93677312 | *TAP2* | 0,2238 | -3,573 to 4,021 | No | ns | >0,9999 |
| Human BSC | 11,0797 | 14,109863 | *TLR3* | 2,61 | -1,186 to 6,407 | No | ns | 0,6218 |
