# Supplementary material for: Transcriptome Profile During Rabies Virus Infection: Identification of Human CXCL16 as a Potential New Viral Target
Source: Front Cell Infect Microbiol. 2021 Nov 5;11:761074. doi: 10.3389/fcimb.2021.761074 (PMC8602097; doi:10.3389/fcimb.2021.761074)
Supplement: Supplementary Figure 1 — Validation of human and murine housekeeping genes (ACTB, GAPDH and 18S). (A) Actb and Gapdh presented the lowest pairwise variation for murine CX and BSC and were subsequently selected as murine housekeeping genes to normalize gene expression of murine target genes. (B) 18S and GAPDH presented the lowest pairwise variation in human BSC biopsies and were subsequently selected as housekeeping genes to normalize gene expression of human target genes. (A, B) Selection of housekeeping genes was performed as described by Vandesompele and colleagues (Vandesompele et al., 2002). Actb, actin beta; BSC, brainstem/cerebellum, CX, cortex; GAPDH, glyceraldehyde-3-phosphate dehydrogenase. [file Presentation_1.zip › Supplementary Material_updated/Table_S5.docx]

**Table S5.** **Identification of differentially expressed genes between CX (n=6) and BSC (n=6) of RABV strain Tha-infected mice.** Gene expression values (ΔΔCT) were calculated based on non-infected control animals (CX [n=6]; BSC [n=6]). Differential gene expression was defined as a significant variation (adjusted p-value < 0.05) of gene expression (ΔΔCT) between murine the Tha-infected CX and the Tha-infected BSC by using the Šídák's multiple comparisons test (**** p-value < 0.0001, *** p-value < 0.001 ** p-value < 0.01, * p-value < 0.05).

| **Gene** | **Mean Difference** | **95,00% CI of diff.** | **Summary** | **Adjusted P Value** |
| --- | --- | --- | --- | --- |
| **Adar** | -9,833E-07 | -1,336 to 1,336 | ns | >0,9999 |
| **B2m** | -4,167E-07 | -1,336 to 1,336 | ns | >0,9999 |
| **C3ar1** | -8,333E-07 | -1,336 to 1,336 | ns | >0,9999 |
| **Ccl3** | -6,833E-07 | -1,336 to 1,336 | ns | >0,9999 |
| **Ccl5** | 0,000006933 | -1,336 to 1,336 | ns | >0,9999 |
| **Cd74** | -0,00000065 | -1,336 to 1,336 | ns | >0,9999 |
| **Cd86** | 0,1197 | -1,216 to 1,456 | ns | >0,9999 |
| **Cx3cr1** | 2,617E-07 | -1,336 to 1,336 | ns | >0,9999 |
| **Cxcl10** | -0,00002567 | -1,336 to 1,336 | ns | >0,9999 |
| **Cxcl12** | -1,817E-07 | -1,336 to 1,336 | ns | >0,9999 |
| **Cxcl14** | 1,617E-07 | -1,336 to 1,336 | ns | >0,9999 |
| **Cxcl16** | 0,000001783 | -1,336 to 1,336 | ns | >0,9999 |
| **Gbp2b** | 4,833E-07 | -1,336 to 1,336 | ns | >0,9999 |
| **H2eb1** | -3,833E-07 | -1,336 to 1,336 | ns | >0,9999 |
| **H2k2** | -0,0000011 | -1,336 to 1,336 | ns | >0,9999 |
| **Ifit2** | 0,000001867 | -1,336 to 1,336 | ns | >0,9999 |
| **Ifnar1** | -6,667E-08 | -1,336 to 1,336 | ns | >0,9999 |
| **Ifngr2** | 3,333E-08 | -1,336 to 1,336 | ns | >0,9999 |
| **Ikbkb** | -7,667E-08 | -1,336 to 1,336 | ns | >0,9999 |
| **IL13ra1** | 4,333E-07 | -1,336 to 1,336 | ns | >0,9999 |
| **Il6** | 0,3712 | -0,9649 to 1,707 | ns | >0,9999 |
| **Irf7** | 3,833E-07 | -1,336 to 1,336 | ns | >0,9999 |
| **Isgf3** | 1,587 | 0,2508 to 2,923 | ** | 0,0059 |
| **Jak2** | 4,833E-08 | -1,336 to 1,336 | ns | >0,9999 |
| **Jun** | 8,167E-07 | -1,336 to 1,336 | ns | >0,9999 |
| **Oasl1** | 0,3473 | -0,9888 to 1,683 | ns | >0,9999 |
| **Rnasel** | -0,8252 | -2,161 to 0,5109 | ns | 0,8133 |
| **Rtp4** | 0,05505 | -1,281 to 1,391 | ns | >0,9999 |
| **Socs3** | 3,095 | 1,759 to 4,431 | **** | <0,0001 |
| **Stat1** | -0,00000165 | -1,336 to 1,336 | ns | >0,9999 |
| **Stat2** | -0,0000003 | -1,336 to 1,336 | ns | >0,9999 |
| **Tap2** | 7,833E-07 | -1,336 to 1,336 | ns | >0,9999 |
| **Tlr3** | 0,000002117 | -1,336 to 1,336 | ns | >0,9999 |
